# Evaluation of source data verification in a multicentre cancer trial (PROTECT)

**DOI:** 10.1186/1745-6215-14-S1-O80

**Published:** 2013-11-29

**Authors:** J Athene Lane, Michael Davis, Elizabeth Down, Rhiannon Macefield, David Neal, Freddie Hamdy, Jenny Donovan, Hilary Taylor

**Affiliations:** 1 University of Bristol, Bristol, UK; 2 University of Cambridge, Cambridge, UK; 3 University of Oxford, Oxford, UK

## Background and aims

Source Data Verification (SDV) aims to assure data quality and participant safety by checking trial data against source data in site monitoring visits. SDV is resource-intensive but its value is unclear. We evaluated SDV in an ongoing phase III RCT of three prostate cancer treatments (ProtecT) with no planned SDV.

## Methods

Two experienced ProtecT Data Managers reviewed 20 randomly selected participants notes at 9 hospital visits across the UK (around 7% participants per site). SDV case report forms (CRFs) were completed using hospital and trial records (blinded to original CRFs) including baseline, eligibility, treatment and annual outcome CRFs. CRFs were entered on a separate database. Staff time, accommodation and travel costs were recorded and analysed using university costing software.

## Results

639 SDV CRFs were completed (mean 4/participant) from 161 sets of available records (90% of those requested) taking a mean of 51 minutes/participant. Problems encountered included the knowledge required to interpret medical records and interrogate computer systems. SDV and original data were compared for concordance, with errors categorised as critical/major/minor. Trial CRFs were subsequently modified to increase standardisation of data collection across sites. Staff time was the major resource (£7,041) as was £3,126 of subsistence/travel costs (total £10,167, £63/participant, with overhead costs £17,480).

## Conclusions

SDV was conducted at 9 site visits on around 5% of participants in a cancer trial. Logistical issues hindered data collection. Comparison of SDV data against trial data helped improved trial CRFs. Further analysis will evaluate data quality gains against the considerable costs.

